# One novel virus, different beliefs as playmakers towards disease spread in Africa: looking at COVID-19 from a religious lens

**DOI:** 10.11604/pamj.2020.36.365.25114

**Published:** 2020-08-28

**Authors:** Edward Kwabena Ameyaw, John Elvis Hagan, Bright Opoku Ahinkorah, Abdul-Aziz Seidu, Thomas Schack

**Affiliations:** 1 The Australian Centre for Public and Population Health Research, Faculty of Health, University of Technology Sydney, Sydney, Australia,; 2 Department of Health, Physical Education and Recreation, University of Cape Coast, Cape Coast, Ghana,; 3 Neurocognition and Action-Biomechanics-Research Group, Faculty of Psychology and Sport Sciences, Bielefeld University, Bielefeld, Germany,; 4 Department of Population and Health, University of Cape Coast, Cape Coast, Ghana,; 5 College of Public Health, Medical and Veterinary Sciences, James Cook University, Townsville, Queensland 4811, Australia

**Keywords:** Africa, Allah, Christianity, COVID-19, God, Islam, religion, spirituality, religious rituals

## Abstract

Religious and spiritual observances that draw large people together are pervasive in many parts of the world, including Africa. With the recent emergence of COVID-19, these mass religious gatherings may pose significant threats to human health. Given the compromised healthcare systems in many parts of Africa, faith-based institutions have a huge responsibility towards the management of the potential spread of the virus through effective organizational strategies or interventions. This essay sheds light on what the novel virus has to do with religion, the role of religious practices in inhibiting or spreading COVID-19, and what appropriate evidence-based interventions religious or faith-based organizations could adopt to help prevent the spread of the disease in Africa through a unity of thoughts for religious action.

## Essay

The coronavirus 2019 (COVID-19) is one of the scariest pandemics the world has ever known since its emergence in Wuhan, China in November, 2019. The spread of the virus in Africa has been exponential since the first case was recorded on February 14, 2020 in Egypt. There are several instances by which the virus can be contracted unconsciously such as interacting with an asymptomatic carrier without ensuring any safety precaution like keeping at least 6 feet (2 meter) distance. Nonetheless, a greater section of the population may contract the virus due to negligence manifested by disregarding the globally acceptable best practices. One´s acceptance and observation of the best practices are hinged on a multitude of factors including convictions and beliefs held by the person. Therefore, the role of religion cannot be underestimated when discussing the spread of COVID-19 in Africa, a region where nearly everyone belongs to a religion and religion has been considered as constituting an inextricable part of African society [1, 2].

Religion serves as a powerful vehicle for indoctrinating, and getting masses of people to behave in a particular way without questioning or weighing the pros and cons of their actions [3]. Whenever harmful diseases occur with minimal understanding of their etiology, the religious narrative in Africa mostly align them to natural or metaphysical happenings [4]. This embodies the thinking that a disease or unpleasant circumstance originated from the spiritual realm including punishment from God, ancestors or witchcraft [2]. These thinking patterns can truncate the efficacy of the “truths” and precautionary measures that are outlined by the global community. As religion and spiritual practices mark the lives of many Africans, teachings of the dominant religions such as Christianity and Islam can craft a worldview about the virus in this regard and thereby compromise initiatives to halt the spread as transpired in the case of the Ebola virus outbreak [5]. The current essay provides an overview by linking the novel virus with religion, the potential impact of religious practices in inhibiting or facilitating the spread of COVID-19, and what appropriate evidence-based interventions religious or faith-based organizations could employ to prevent the spread of the disease in Africa.

**Potential spread of COVID-19 through religious gatherings:** religious and/or spiritual orientation and finding time for related behaviours are pervasive and structure the lives of nationals from the sub-region. People´s religious itinerary involves moving back and forth with dual or multiple affiliations, and that religious practices vary according to context or specific needs [6, 7]. Allegiance to a particular religious affiliation becomes more identifiable in situations when specific individuals or groups´ needs are met and vice versa. Notwithstanding the prevalence of belief in God, Allah or a deity, not all people believe, and believers vary markedly due to their faith and as well have variance in religious practice between the public and private realm [7, 8]. According to some authors [9], these variations are perhaps due to cultural transmission, where individuals´ beliefs are affected by their social contexts and cognitive style. For example, worshipping is predominantly on Sundays by Christians in churches, with Muslims on Fridays in mosques, while visiting shrines for spiritual consultations are exclusively secret (i.e. kept away from the public eyes) [9, 10].

Religious and faith-based gatherings could provide an avenue for the potential spread of the coronavirus. COVID-19 is transmittable through tiny respiratory droplets when an infected person sneezes, coughs, or talks as well as gets in touch with contaminated surfaces [11]. These tiny droplets can get to people or be inhaled by other persons nearby. Amidst COVID-19, Christians earmarked Easter during the second week of April, 2020, an annual religious commemoration event that heralds the death and resurrection of Christ through activities such as the entrance to Jerusalem, the last supper, the *viacrucis*, the death and resurrection of Jesus of Nazareth [12]. The liturgies and related religious activities often attract large crowds of various Christian denominations across many countries, including those in Africa. Similarly, majority of Muslims around the world, including Africa commenced their annual fasting on April 24, often described as Holy Month of Ramadan, a period noted for prayers, worship, comradeship, and building relationships. The climax of Ramadan comprises a celebration called Eid al-Fitr, when the traditional month-long fast is ended with a feast. All these mass religious gatherings could facilitate the “super-spreading” of the virus if precautionary measures are not taken. Many of these faith observances (e.g. prayers) comprise physical contact (i.e. holding hands) between their worshippers. Therefore, the tiny respiratory droplets containing the virus could settle on a person´s hands and be transferred to others through physical contact. Other religious practices include touching or kissing of sacred and symbolic objects during worship services and prayers. The causative organism of COVID-19 could then stay on such surfaces for hours or days [13]. Hence, potential cases of COVID-19 may be scattered and transferred out of different religious practices through local transmissions into communities, national, and regional level across Africa.

The notion held about the virus will eventually dictate the control or curative measures to be taken [2]. It also affects the extent to which the evidence-based precautionary measures will be observed. For example, during the Ebola outbreak in Africa, several traditional and spiritual healers falsely claimed to have the capability to cure the disease [14]. Other media reportage indicated that many Christian and Muslim leaders prayed for Ebola victims and in the process contracted the disease and/ or transferred it to other people [2]. Similar to Ebola, there have been some media reports in Africa that some pastors and other religious leaders gathered their members to pray against COVID-19 when the first cases of the virus were recorded on the continent. There are reported cases of defiance by some churches who occasionally defied mitigating strategies (e.g. social distancing) outlined by governments in fighting COVID-19. The risks of such actions by these religious organizations threaten everyone beyond their congregational boundaries and their mass congregational services and offer an effective platform for quickening the virus spread [15]. Given the enormous challenge confronting Africa in the fight against the virus, religious institutions and faith-based organizations have a huge responsibility to protect their numerous followers through interventions that recognize personal safety.

**Managing COVID-19 by faith-based institutions:** while admitting that obstinate religious congregations can fast-track the viral transmission, religious leaders, faith-based organizations, and faith communities can also play a key role in protecting the masses and further reduce the disease burden associated with COVID-19. These religious organizations can also be the main sources of support, comfort, guidance, and direct healthcare and social service for the local communities they work for [13]. According to Wildman, Bulbulia, Sosis and Schjoedt [15], most religious groups can offer more innovative means of reaching out to the followers in response to the challenging demands of collective congregational gatherings. For instance, faith-based organizations and religious communities in other societies are delivering online religious services by using video as well as audio conferencing and virtual gatherings for their sermons and prayers. Religious leaders can use the same platforms to disseminate practical health information (e.g. frequent hand washing, use of hand sanitizers, physical distancing) related to COVID-19 to offer protection for their own members and wider communities, including vulnerable populations. By providing clear, simple and practical steps to prevent COVID-19, these religious institutions would be helping in alleviating fear and stigma, while providing encouragement and health-promoting behaviours to people in their communities [13].

WHO has reiterated that these religious organizations are deeply integrated into local communities through service and other kindhearted networks through their sub-groups that are tasked to identify most vulnerable populations requiring urgent assistance. Hence, religious leaders may serve as useful links for providing personal safety for vulnerable persons within their communities and beyond. The following guidance and recommendations have been developed by the WHO in response to the COVID-19 pandemic regarding the operations of religious and faith-based organizations: observing at least 1m (3 feet) of distance between people at all times; preventing touching between people attending faith services of any kind; preventing touching or kissing of devotional and other objects that followers are accustomed to use for communal purposes; promoting healthy hygiene among members during faith services and other activities when gatherings are permitted; regularly clean worship spaces, sites, and buildings with appropriate detergents or disinfectants [13].

Beyond these practical evidence-based steps, religious organizations can also provide financial assistance to compliment donations from other corporate institutions to augment state resources in the aftermath of worsening economic conditions globally. While admitting global diversity among religious entities, many of these organizations are directly involved with the battle against COVID-19, an indication of their social and corporate responsibility during health and other emergencies such as the current pandemic. More scientific enquiry into the impact of religion can help provide much empirical linkages between transcendence and human behaviours related to health. Opening an empirical investigation to test the effectiveness of religious coping against risk perceptions and vulnerabilities related to COVID-19 would be very useful.

**Conclusions:** even though epidemiological models of virus transmission fail to recognize human factors like religious ideologies and values [13], “religion is the opium of the people” regardless of its complexities [12]. Therefore, an eclectic approach that takes into account religious practices and teachings may be a more promising strategy for Africa to help overcome the COVID-19 pandemic. At the sub-regional, national and community levels, religious leaders should therefore be actively engaged while following scientific truths and empirically-driven parameters about the causation and prevention of the disease. Recognizing religious collaborations would be very crucial towards the dissemination of health information, resources, and appropriate practices where possible in order to help prevent and/ or minimize the spread of the virus in Africa.
